# Shedding Kinetics and Infection Stage-Dependent Horizontal Transmission of Infectious Spleen and Kidney Necrosis Virus (ISKNV) in Angelfish (*Pterophyllum scalare*)

**DOI:** 10.3390/ani16172751

**Published:** 2026-09-02

**Authors:** So-Won Choi, Ye-Jin Jeong, Eun-Jin Baek, Woo-Ju Kwon, Chonghan Kim, Kwang-Il Kim

**Affiliations:** 1 Department of Aquatic Life Medicine, Pukyong National University, Busan 48513, Republic of Korea; thdnjs3688@gmail.com (S.-W.C.); 201513346@pukyong.ac.kr (Y.-J.J.); eungene8803@gmail.com (E.-J.B.); 2 Vaccine Research Institute, Woogene B&G Co., Ltd., Seoul 07299, Republic of Korea

**Keywords:** *Megalocytivirus pagrus* I, infectious spleen and kidney necrosis virus, horizontal transmission, waterborne transmission, angelfish

## Abstract

Infectious spleen and kidney necrosis virus (ISKNV), a member of the genus Megalocytivirus, threatens ornamental and farmed aquaculture because of its broad host range and environmental persistence. This study investigated ISKNV infection dynamics and stage-dependent horizontal transmission in angelfish (*Pterophyllum scalare*). Immersion challenge experiments demonstrated that angelfish were highly susceptible to ISKNV infection, with cumulative mortality reaching 95% at 10^6^ TCID_50_/mL. The estimated LD_50_ and ID_50_ values indicated that infection occurred even at sublethal viral concentrations. Viral loads peaked at 7 days post-immersion and were positively correlated with the severity of histopathological lesions in the spleen and kidney, including multifocal necrosis and enlarged basophilic cells. To assess horizontal transmission, naive fish were cohabited with infected donors for 24 h at different infection stages. These findings indicate that ISKNV transmission can occur following short-term cohabitation and that transmission outcomes may vary according to the infection stage of donor fish under the experimental conditions examined, providing insights into ISKNV transmission dynamics and the potential implications for disease management in the ornamental fish industry.

## Simple Summary

ISKNV is an important viral pathogen that causes severe disease and high mortality in ornamental and cultured fish. Although ISKNV is known to spread through water, little is known about when infected fish are most infectious. In this study, we investigated the progression of ISKNV infection and waterborne transmission using angelfish as a model ornamental fish. The virus rapidly accumulated in the spleen and kidney, causing severe tissue damage as infection progressed. Healthy fish became infected after only a short period of cohabitation with infected fish, with greater recipient mortality observed when donor fish had higher viral loads. These findings provide new insights into ISKNV infection and transmission dynamics in angelfish and suggest that short-term exposure to infected fish may represent a potential transmission risk.

## 1. Introduction

The international ornamental fish market has experienced consistent growth, driven by renewed recognition of its industrial and societal significance. As the ornamental fish trade expands into a global business, it becomes increasingly prone to disease transmission among trading nations [1]. The worldwide commerce of live ornamental fish may have led to the widespread distribution of megalocytiviruses [2,3,4].

Among viruses identified in fish, *Megalocytivirus* has had significant economic and ecological impacts on aquaculture systems producing ornamental species and fish for human consumption [3,5]. The genus *Megalocytivirus* belongs to the family *Iridoviridae* and comprises large icosahedral DNA viruses measuring 120–200 nm in diameter with a large single linear dsDNA [6]. According to the International Committee on Taxonomy of Viruses (ICTV, 2024) [7], *Megalocytivirus* species are currently classified into *Megalocytivirus pagrus* I and *Megalocytivirus lates* I, primarily based on the sequence identity of the major capsid protein (MCP) and adenosine triphosphatase (ATPase) genes. Representative viruses include red sea bream iridovirus (RSIV), infectious spleen and kidney necrosis virus (ISKNV), turbot reddish body iridovirus (TRBIV), and scale drop disease virus (SDDV) [6,8]. Based on their phylogeny, ISKNV and RSIV can be further classified into two subgenotypes [6]. ISKNV-I is prevalent in Thailand, whereas ISKNV-II has been identified in Vietnam [9,10,11].

ISKNV was first reported in Chinese mandarin fish (*Siniperca chuatsi*), which exhibited clinical signs such as anorexia, abnormal swimming, faded body pigmentation, and pale gills [12]. Over the past two decades, ISKNV infections were largely confined to several Asian countries, particularly China, Singapore, Taiwan, Malaysia, Indonesia, and India [1,13]. More recently, its distribution has expanded globally, with reports from Australia, Germany, the United States, and Africa [13,14,15,16]. Because ISKNV has a broad host range and can infect nearly 50 different freshwater, brackish, and marine species [13], its global spread is biologically plausible. Ornamental fish, including cichlids, livebearers, and gourami species, are highly susceptible to ISKNV infection [14].

Horizontal transmission through contaminated water is considered a major route of ISKNV dissemination in aquaculture systems. Infected fish can release large quantities of virus into the surrounding environment, particularly during the acute stage of infection, thereby increasing the risk of transmission to naïve fish populations [5,17]. Environmental viral loads have also been associated with transmission efficiency during megalocytivirus outbreaks [18]. However, quantitative information regarding viral shedding kinetics and stage-dependent transmission dynamics remains limited in ornamental freshwater fish species, highlighting the need for a better understanding of waterborne transmission to inform effective biosecurity management.

Angelfish (*Pterophyllum scalare*) is one of the most widely imported and distributed species in the global ornamental fish trade. It is considered a susceptible host to ISKNV, with natural infections reported in several countries, including the United Kingdom, Singapore, Malaysia, Brazil, India, and Thailand [19,20,21,22,23,24,25]. Despite this susceptibility, comprehensive data on ISKNV pathogenicity and the potential for experimental horizontal transmission in this species remain limited.

This study aimed to evaluate immersion infection and short-term cohabitation models to investigate ISKNV infection dynamics in angelfish under freshwater conditions. Specifically, this study aimed to (1) determine the dose-dependent pathogenicity and infectivity of ISKNV in angelfish following immersion exposure, (2) characterize temporal viral replication and associated histopathological progression in infected tissues, and (3) quantify viral shedding and evaluate stage-dependent horizontal transmission during short-term cohabitation, thereby providing insights into ISKNV transmission dynamics and the potential implications for disease management in the ornamental fish industry.

## 2. Materials and Methods

### 2.1. Experimental Fish and Virus

Healthy angel fish (mean body length, 4.8 ± 0.71 cm; mean body weight, 1.79 ± 0.79 g) were bred and maintained in our laboratory. Fish were maintained in 100 L freshwater tanks at 28 °C, with approximately 20% of the water replaced daily using temperature-adjusted freshwater (28 ± 0.5 °C). The fish were fed a commercial diet once daily. All experiments were conducted after two randomly selected angelfish were confirmed to be negative for ISKNV by polymerase chain reaction (PCR), as described by Kurita et al. [26].

The ISKNV strain DGIV-SAY-23 (GenBank accession No. OR822346), originally isolated in 2024 from dwarf gourami (*Trichogaster lalius*) imported from Singapore to the Republic of Korea, was used in this study [27]. The virus was propagated in the dwarf gourami fin cell line [28], which was cultured at 28 °C in Leibovitz’s L-15 medium (Gibco, New York, NY, USA) supplemented with 5% heat-inactivated fetal bovine serum (FBS; 55 °C for 30 min) and 1 × gentamicin (Welgene, Gyeongsan, Republic of Korea). Cell culture supernatants were harvested upon the appearance of cytopathic effects (CPE), typically at 7 days post-inoculation. The harvested supernatants underwent three freeze–thaw cycles and were centrifuged at 500× *g* for 10 min at 4 °C. Subsequently, the clarified supernatant was collected, and infective titers and viral genome copy numbers were determined using the 50% tissue culture infectious dose (TCID_50_) assay [29] and quantitative PCR (qPCR) [30], respectively. The prepared viral stocks were then stored at −80 °C until use.

### 2.2. Dose-Dependent Immersion Infection in Angelfish

**Figure 1:**
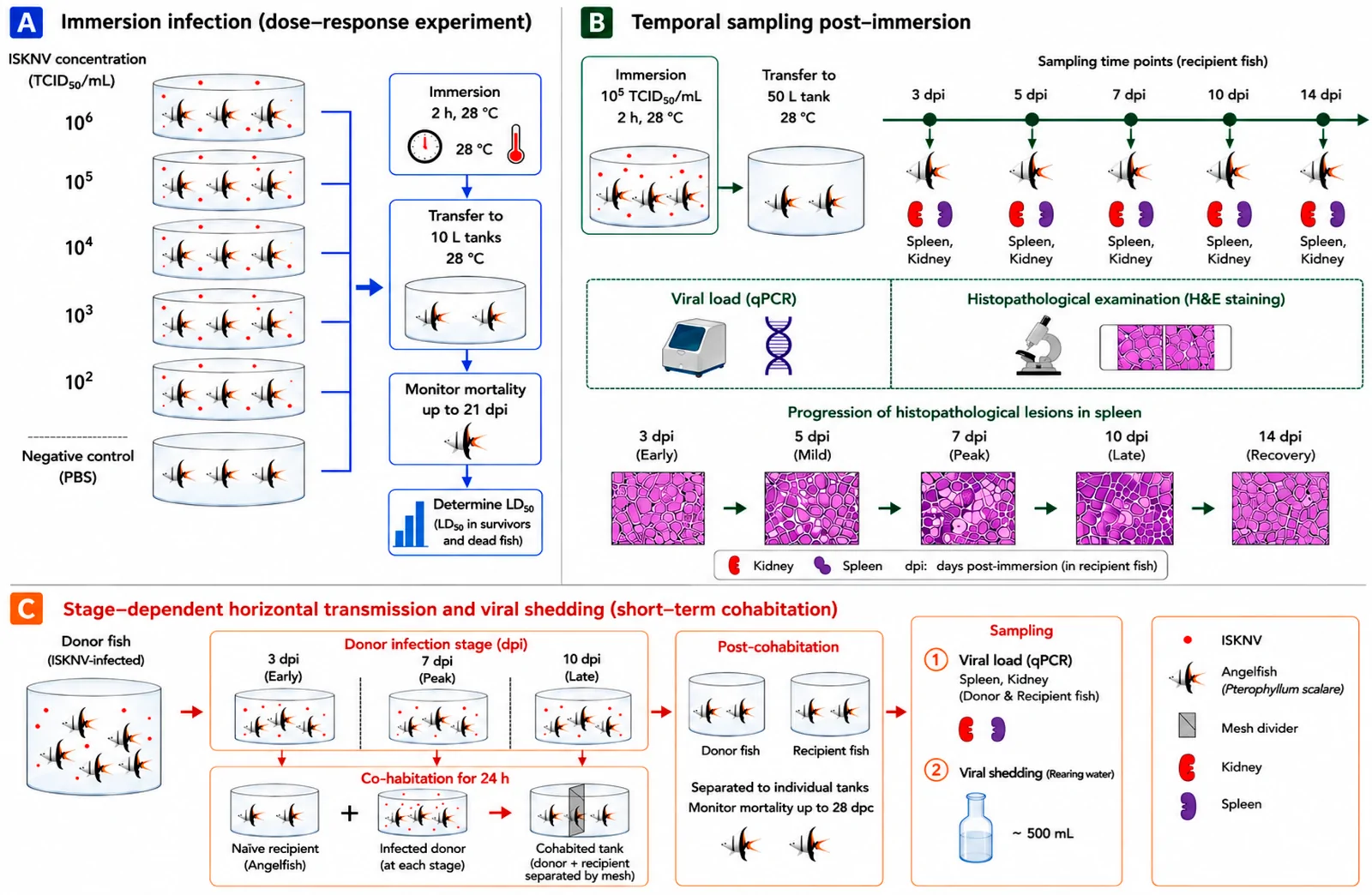
Schematic diagram of the workflow. (**A**) Immersion exposure of angelfish to infectious spleen and kidney necrosis virus (ISKNV) at 28 °C using five viral concentrations (10^6^, 10^5^, 10^4^, 10^3^, and 10^2^ ISKNV TCID_50_/mL). (**B**) Sampling of spleen and kidney tissues at 3, 5, 7, 10, and 14 days post-immersion (dpi) to evaluate the viral load and histopathological lesions. (**C**) Quantification of viral shedding following 24 h cohabitation exposure, highlighting the associated risk of horizontal transmission.

Angelfish (*n* = 10 per treatment group in each replicate) were exposed to ISKNV at final concentrations of 10^2^, 10^3^, 10^4^, 10^5^, and 10^6^ TCID_50_/mL by immersion for 2 h at 28 °C. Following exposure, fish were transferred to separate 10 L tanks containing fresh rearing water and maintained at 28 °C. The negative control group (*n* = 10 per replicate) was immersed in freshwater containing an equivalent volume of phosphate-buffered saline (PBS). All fish were maintained under identical conditions in 10 L tanks at 28 °C, with 50% daily water exchange and feeding twice daily with a commercial diet. Mortality was monitored for 21 days post-immersion (dpi) to estimate the median lethal dose (LD_50_). Additionally, the median infection dose (ID_50_) was evaluated in both dead and surviving fish at 21 dpi across all exposure concentrations. The immersion challenge experiment was conducted as two independent biological replicates performed at different time points under identical experimental conditions, with 10 fish per treatment group in each replicate (*n* = 10 per replicate; total *n* = 20 per treatment group). Data from the two independent replicates were combined for cumulative mortality. All animal experiments were performed according to the guidelines of the Animal Research and Ethics Committee of Pukyong National University (approval no. PKNUIACUC-2025-30). Figure 1A illustrates a schematic overview of the experimental design.

### 2.3. Viral Kinetics and Pathological Staging Following Immersion Infection

To evaluate the viral load dynamics of ISKNV infection in angelfish, healthy fish (*n* = 30) were exposed to the DGIV-SAY-23 strain of ISKNV at a concentration of 10^5^ TCID_50_/mL by immersion for 2 h at 28 °C. After exposure, the fish were transferred to a separate 50 L tank containing fresh water. The control group (*n* = 30) was exposed to PBS alone. For viral load analysis, spleen and kidney tissues were collected from three individual fish randomly sampled from the same experimental tank at 3, 5, 7, 10, and 14 dpi and analyzed using qPCR. Furthermore, to assess histopathological lesions over the course of ISKNV infection, three individual fish were sampled from the experimental tank at 3, 5, 7, 10, and 14 dpi and fixed in Bouin’s solution for 24 h. Following fixation, samples were transferred to 70% ethanol overnight, dehydrated through a graded ethanol series (70%, 80%, 85%, 90%, 95%, and absolute ethanol), cleared in xylene, and embedded in paraffin. Once embedded in paraffin blocks, serial cross-sections (4 μm) were obtained by cutting the fish from head to tail using a rotary microtome (RM2125RT, Leica Biosystems, Wetzlar, Germany) and stained with hematoxylin and eosin. Histopathological evaluation focused on the spleen and kidney. Sections were examined qualitatively under an optical microscope (BX50, Olympus Ltd., Tokyo, Japan), and images were captured using a digital camera (DP72, Olympus, Tokyo, Japan). Histopathological scores were determined using the grading system described by Kim et al. [31]. The lesion severity associated with ISKNV infection was categorized into four stages: (1) less severe, (2) mild, (3) moderate, and (4) severe. The infected lesions were classified into two categories: enlarged cells and necrotic lesions. The lesions observed in the spleen and kidney tissues were assessed and integrated into a severity score based on the extent of enlarged cell formation and tissue necrosis. A score of 1 (less) indicated that melano-macrophage centers (MMCs) were localized within tissues with minimal focal necrosis, and a score of 2 (mild) indicated that a limited number of enlarged cells were observed in the renal tubules, parenchymal tissues, or glomeruli. A score of 3 (moderate) indicated the multifocal presence of enlarged cells with evident tissue degeneration, and a score of 4 (severe) indicated diffuse distribution of enlarged cells and extensive necrotic lesions, including parenchymal atrophy. In addition, a score of 0 (normal) was assigned when no histopathological lesions were observed and tissue morphology was comparable to that of the control group. Figure 1B provides a schematic overview of the experimental design.

### 2.4. Stage-Dependent Horizontal Transmission via Cohabitation

A cohabitation experiment was conducted to evaluate stage-dependent horizontal transmission of ISKNV. Donor fish (*n* = 10 per group) were immersed in an ISKNV suspension of 10^5^ TCID_50_/mL for 2 h at 28 °C. At 3, 7, and 10 days post-immersion (dpi), naive recipient fish (*n* = 10 per group) were cohabited with donor fish for 24 h in the same 10 L tank. Donor and recipient fish were housed in separate mesh cages within the same tank to allow free water exchange while preventing direct physical contact. Following the 24 h cohabitation period, the donor and recipient fish were transferred to separate 10 L tanks. For the negative control group, angelfish were immersed in PBS instead of ISKNV and cohabited with naive fish under the same conditions. All groups were maintained at 28 °C, with 50% daily water exchange, and were fed twice daily. Cumulative mortality was observed for each group over 28 days. To assess ISKNV infection in dead and surviving fish, spleen and kidney tissues were collected and analyzed using qPCR. Furthermore, to quantify viral shedding from the donor fish into the environment, three 500 mL water subsamples were collected from the same experimental tank following the 24 h cohabitation period at each donor infection stage (3, 7, and 10 dpi). Each cohabitation treatment was conducted in a single tank, which served as the experimental unit. Figure 1C provides a schematic overview of the experimental design.

### 2.5. Quantification of Viral Shedding in Rearing Water Using Aluminum Flocculation

The aluminum flocculation method was used to detect and quantify ISKNV particles released by donor fish into rearing water at 3, 7, and 10 dpi, following the protocol described by Kim et al. [32]. A total of 500 mL of rearing water was filtered through a 1.6 μm pore size glass microfiber filter (GF/A; Whatman, Maidstone, UK) to remove suspended particles. To generate aluminum-virus flocculates, 6.34 g of aluminum sulfate [Al_2_(SO_4_)_3_·6H_2_O] was dissolved in 50 mL of distilled water, and 50 μL of the resulting solution was added to 500 mL of freshwater. Fresh water containing the aluminum-sulfate solution was gently stirred at room temperature for 30 min at 120 rpm using a magnetic stirrer. After 30 min, the Al-ISKNV flocculates were collected by filtering through a polycarbonate membrane (pore size: 1.0 µm; Whatman, Maidstone, UK) using a peristaltic pump at a pressure of <15 psi (Eyela, Tokyo, Japan). The membrane containing the flocculated virus was transferred to a 5 mL round-bottom tube, and 2 mL of elution buffer (0.1 M Tris, 0.1 M EDTA·6H_2_O, 0.2 M MgCl_2_·6H_2_O, 0.2 M L-ascorbate; pH 6.0; Sigma-Aldrich, St. Louis, MO, USA) was added. Viral particles were resuspended using a BioRS-24 Mini-Rotator (Biosan, Riga, Latvia) at 30 rpm for 2 h at 4 °C in the dark. To quantify viral genome copies in the rearing water concentrate, total DNA was extracted from 200 μL of the viral resuspended buffer using a yesG Cell Tissue Mini Kit (Gene Gen, Busan, Republic of Korea), and quantitative analysis was performed using qPCR. To determine the viral shedding rate in ISKNV-infected donor angelfish, the viral genome copy number per liter of water was normalized to the mean body weight of the fish in each group and expressed as genome copies/L/g/day.

### 2.6. Polymerase Chain Reaction and Quantitative Polymerase Chain Reaction Assays

ISKNV was detected using conventional PCR as described by Kurita et al. [26]. Viral genome copies were determined using qPCR, as described by Kim et al. [30]. Briefly, total genomic DNA was extracted from 10 mg of spleen and kidney tissue using the yesG Cell Tissue Mini Kit (Gene Gen, Busan, Republic of Korea) according to the manufacturer’s protocol. The extracted DNA served as the template for conventional PCR and qPCR assays. Conventional PCR was conducted with a total volume of 20 µL containing 10 µL of Taq polymerase (Prime Taq Premix, Daejeon, Republic of Korea), 1 µL of DNA template, 7 µL of nuclease-free water, and 1 µL of each primer set (1F and 1R). qPCR was performed with a 20 μL final volume reagent mixture containing 1 μL of DNA template, 200 nM of each primer (RSIV 1094F and RSIV 1221R) and probe (RSIV 1177 probe), 10 μL of 2 × HS Prime qPCR Premix (Genet Bio, Nonsan, Republic of Korea), 0.4 μL of 50 × ROX dye, and 7.1 μL of nuclease-free water, using a StepOne Real-time PCR system (Applied Biosystems, Waltham, MA, USA). Table 1 presents the PCR primers and conditions used in this study.

### 2.7. Statistical Analysis

Statistical analyses were performed using GraphPad Prism version 10.6.1 (GraphPad Software, San Diego, CA, USA). Viral genome copy numbers in the spleen and kidney tissues of angelfish collected at 3, 5, 7, 10, and 14 dpi were analyzed using one-way analysis of variance (ANOVA) followed by Dunnett’s multiple comparison test. The correlation between histopathological infection grade and viral load was analyzed using Pearson’s correlation coefficient. Cumulative mortality was analyzed using the Kaplan–Meier survival method, and differences among experimental groups were evaluated using the log-rank (Mantel–Cox) test. Mortality data were fitted to a logistic regression model to estimate LD_50_ (%) and ID_50_ (%). Differences in viral loads according to exposure concentration and survival status were analyzed using two-way ANOVA. All quantitative data are presented as mean ± standard deviation, and statistical significance was set at *p* < 0.05 (* *p* < 0.05; ** *p* < 0.01; *** *p* < 0.001; **** *p* < 0.0001).

## 3. Results

### 3.1. Dose-Dependent Immersion Infection and Mortality Following Immersion Challenge

**Figure 2:**
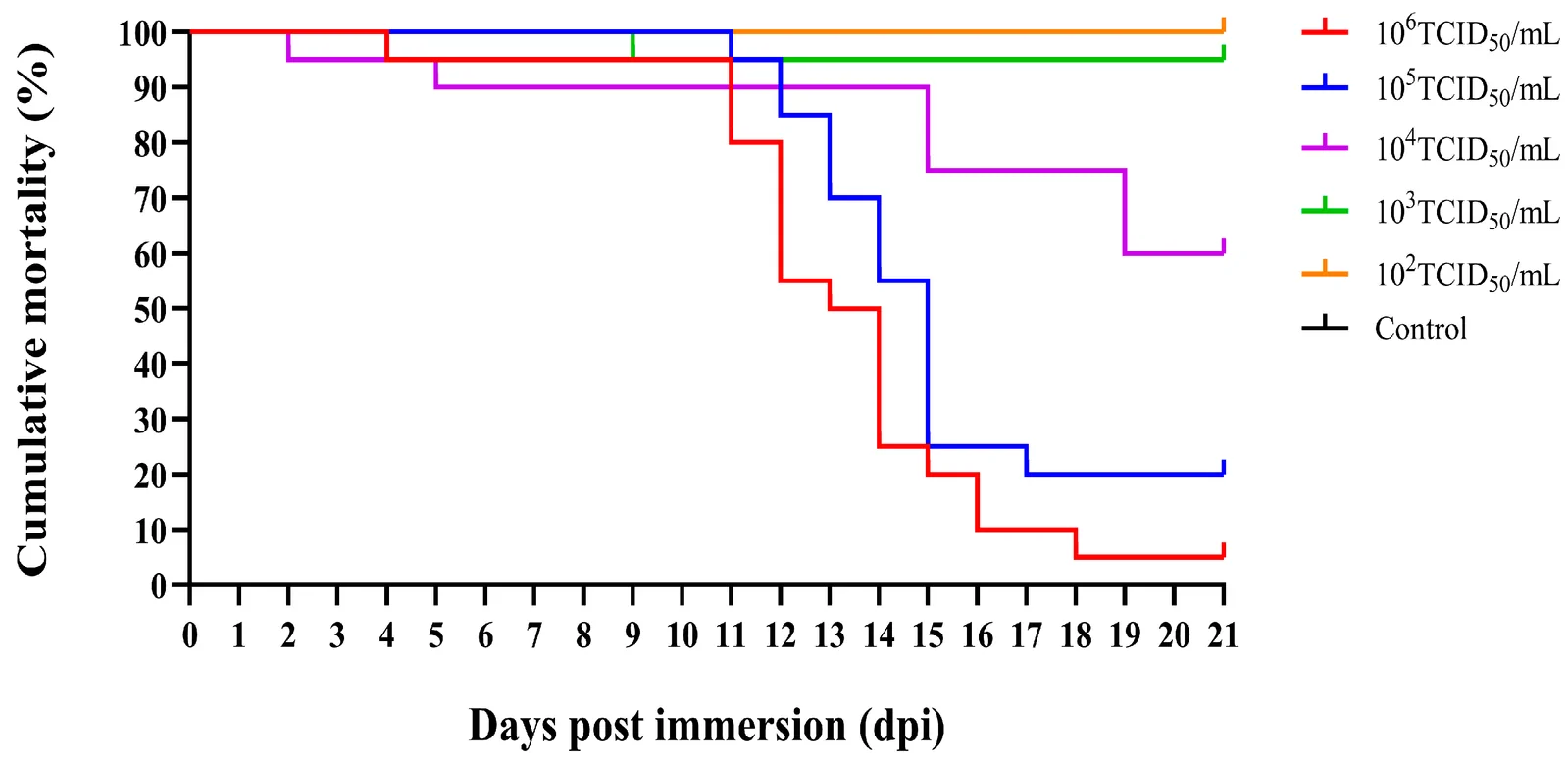
Cumulative mortality of angelfish following immersion challenge exposure to infectious spleen and kidney necrosis virus (ISKNV) at 28 °C was determined using five viral concentrations (10^6^, 10^5^, 10^4^, 10^3^, and 10^2^ ISKNV TCID_50_/mL) and a negative control group during a 21-day post-immersion (dpi). Survival curves were generated using Kaplan–Meier analysis, indicating a significant dose-dependent effect of viral concentration on mortality (log-rank test, *p* < 0.0001).

**Figure 3:**
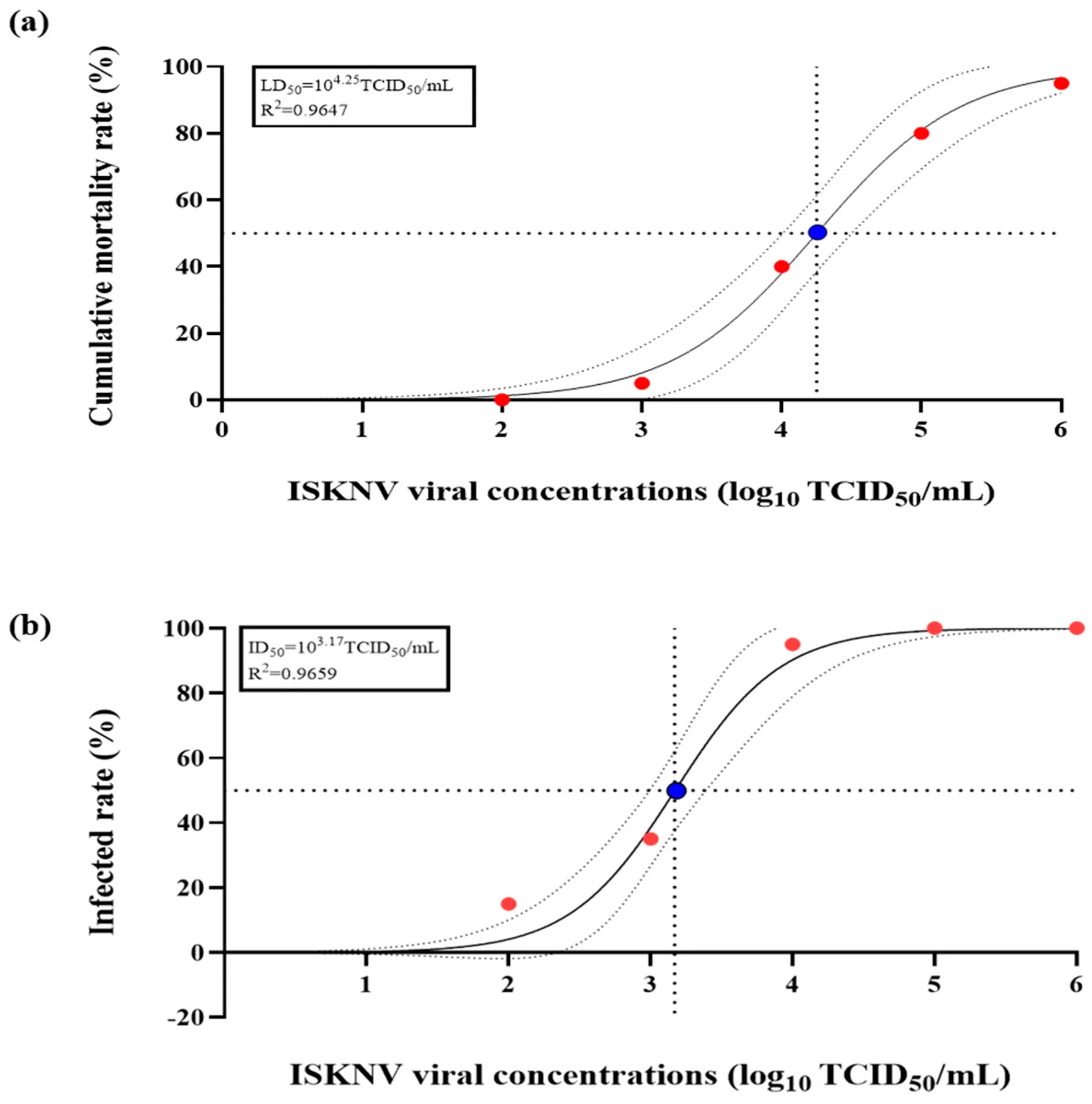
Determination of median lethal dose (LD_50_) and median infection dose (ID_50_) of infectious spleen and kidney necrosis virus (ISKNV) in angelfish following immersion challenge using four-parameter logistic models. (**a**) Cumulative mortality was fitted to a four-parameter logistic regression model (R^2^ = 0.9647), yielding an LD_50_ of 10^4.25^ TCID_50_/mL. (**b**) Infection probability was analyzed (R^2^ = 0.9659), with an ID_50_ of 10^3.17^ TCID_50_/mL. The blue dots and dotted lines indicate the estimated LD_50_ and ID_50_ values for the respective curves.

**Figure 4:**
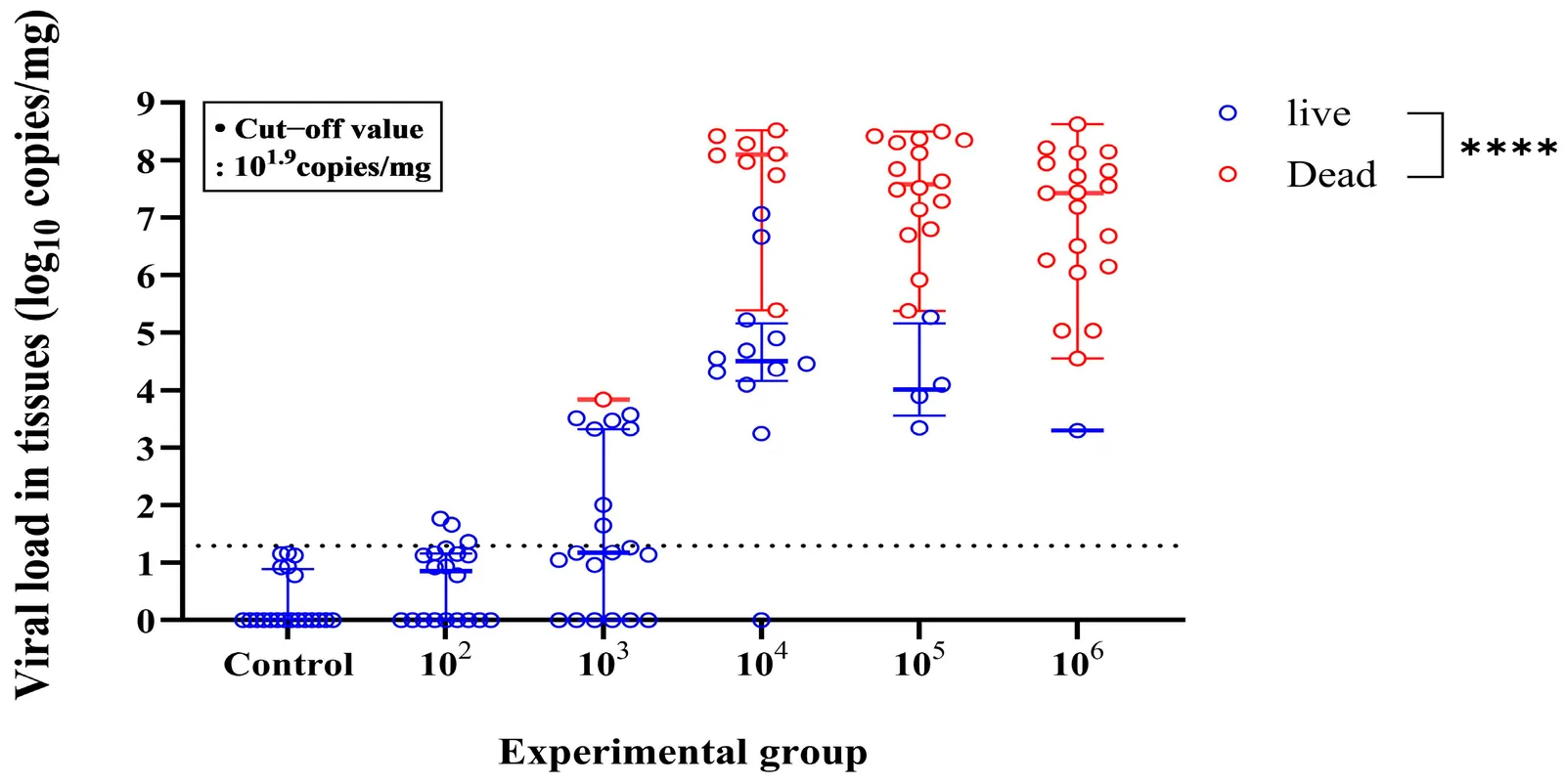
Viral load distribution in angelfish following immersion challenge with infectious spleen and kidney necrosis virus (ISKNV). Infection rates at 21 days post-immersion (dpi) are shown for groups exposed to 10^2^–10^6^ TCID_50_/mL, whereas no infection was detected in the control group. Viral loads in the spleen and kidney are presented for infected, surviving, and dead fish, with each dot representing an individual fish. Horizontal bars indicate mean ± standard deviation (SD), the dotted line denotes the cutoff value (10^1.9^ copies/mg), and percentages above the plots represent the infection rates in each group. Statistical analysis was performed using two-way analysis of variance (ANOVA), revealing a significant effect between live and dead fish groups (*p* < 0.0001).

Dose-dependent ISKNV immersion infection in angelfish was evaluated. Cumulative mortality rates were 95%, 80%, 40%, and 5% in the 10^6^, 10^5^, 10^4^, and 10^3^ TCID_50_/mL groups, respectively, whereas no mortality was observed in the 10^2^ TCID_50_/mL and negative control groups (Figure 2). The calculated LD_50_ (%) for ISKNV immersion infection in angelfish was estimated to be 10^4.25^ ISKNV TCID_50_/mL, indicating a strong correlation with mortality (R^2^ = 0.9647) (Figure 3a). ISKNV infection in dead and surviving fish from each challenged group was confirmed by conventional PCR (Supplemental Figure S1). Infection rates were estimated based on viral loads detected in dead and surviving fish exposed to different ISKNV concentrations. The ID_50_(%) was estimated to be approximately 10-fold lower than the LD_50_ (%), and exposure to 10^3.17^ ISKNV TCID_50_/mL for 2 h was predicted to infect more than 50% of fish (Figure 3b). Furthermore, qPCR analysis demonstrated a concentration-dependent increase in both infection rate and viral genome copy number at 21 dpi following ISKNV exposure (Figure 4). Viral load analysis revealed relatively low infection rates of 15% and 35% in the 10^2^ and 10^3^ TCID_50_/mL exposure groups, respectively, indicating light infection at lower exposure concentrations. In contrast, infection rates increased markedly to 95%, 100%, and 100% in the 10^4^, 10^5^, and 10^6^ TCID_50_/mL groups, respectively, whereas no ISKNV infection was detected in the negative control group.

### 3.2. Temporal Infection Dynamics of Infectious Spleen and Kidney Necrosis Virus in Angelfish

**Figure 5:**
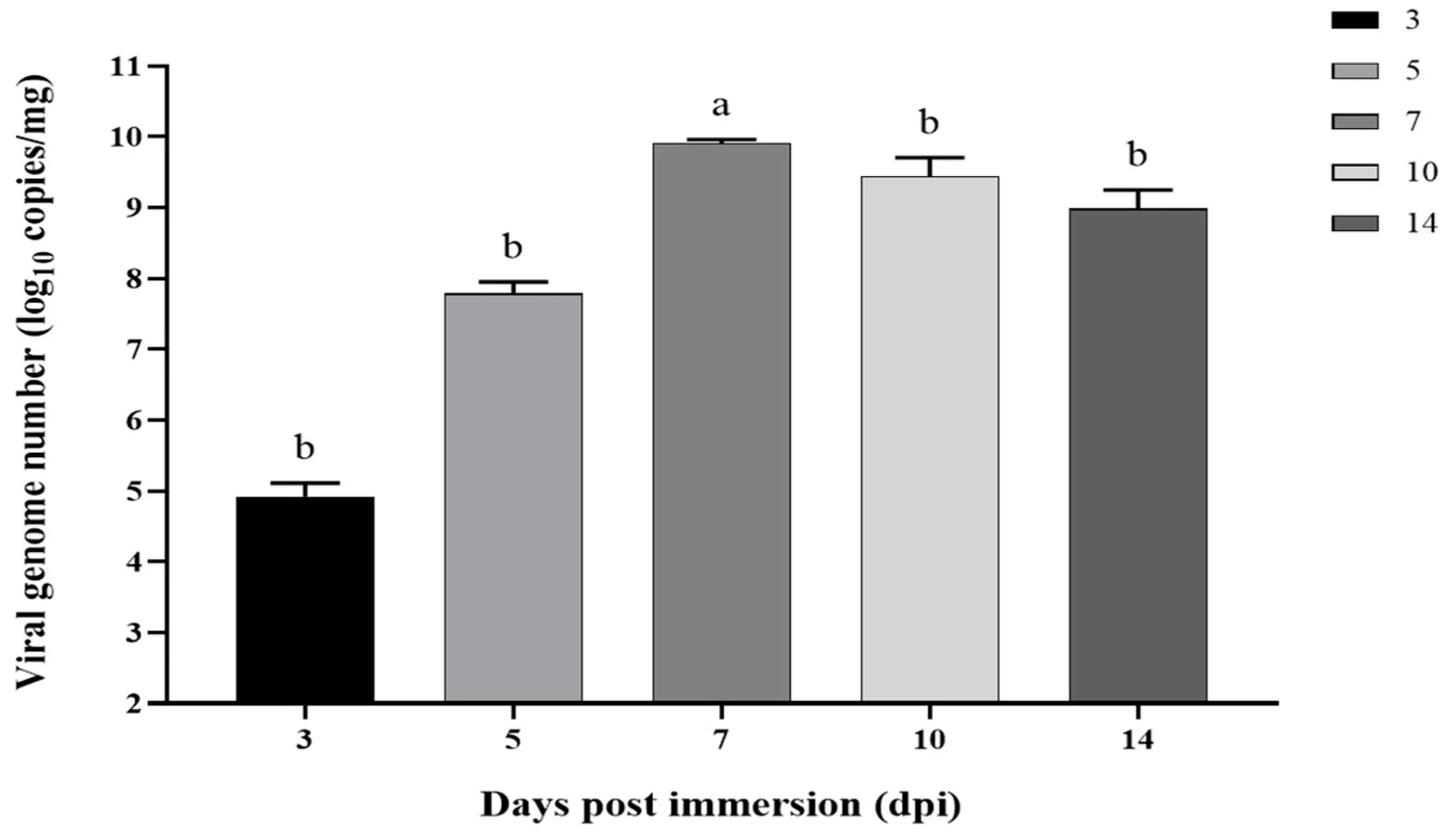
Temporal analysis of viral genome copy numbers in the spleen and kidney of angelfish collected at 3, 5, 7, 10, and 14 days post-immersion (dpi). Data are presented as mean ± standard deviation (SD) of three individual fish sampled from the same experimental tank at 3, 5, 7, 10, and 14 dpi. Statistical analysis was performed using one-way ANOVA followed by Tukey’s multiple comparisons test. Different lowercase letters indicate significant differences among sampling time points (*p* < 0.05).

Temporal infection dynamics in angelfish following ISKNV immersion were analyzed. Viral load increased during the infection period, reaching its highest observed level at 7 dpi. At 3 dpi, viral load reached 8.36 × 10^4^ copies/mg and increased continuously, peaking at 8.28 × 10^9^ copies/mg at 7 dpi. At 10–14 dpi, a slight decrease in viral load was observed. At the end of 14 days, the viral load decreased by approximately 9-fold relative to the peak measured at 7 dpi, reaching 9.78 × 10^8^ copies/mg (Figure 5).

### 3.3. Histopathological Progression Associated with Viral Replication

**Figure 6:**
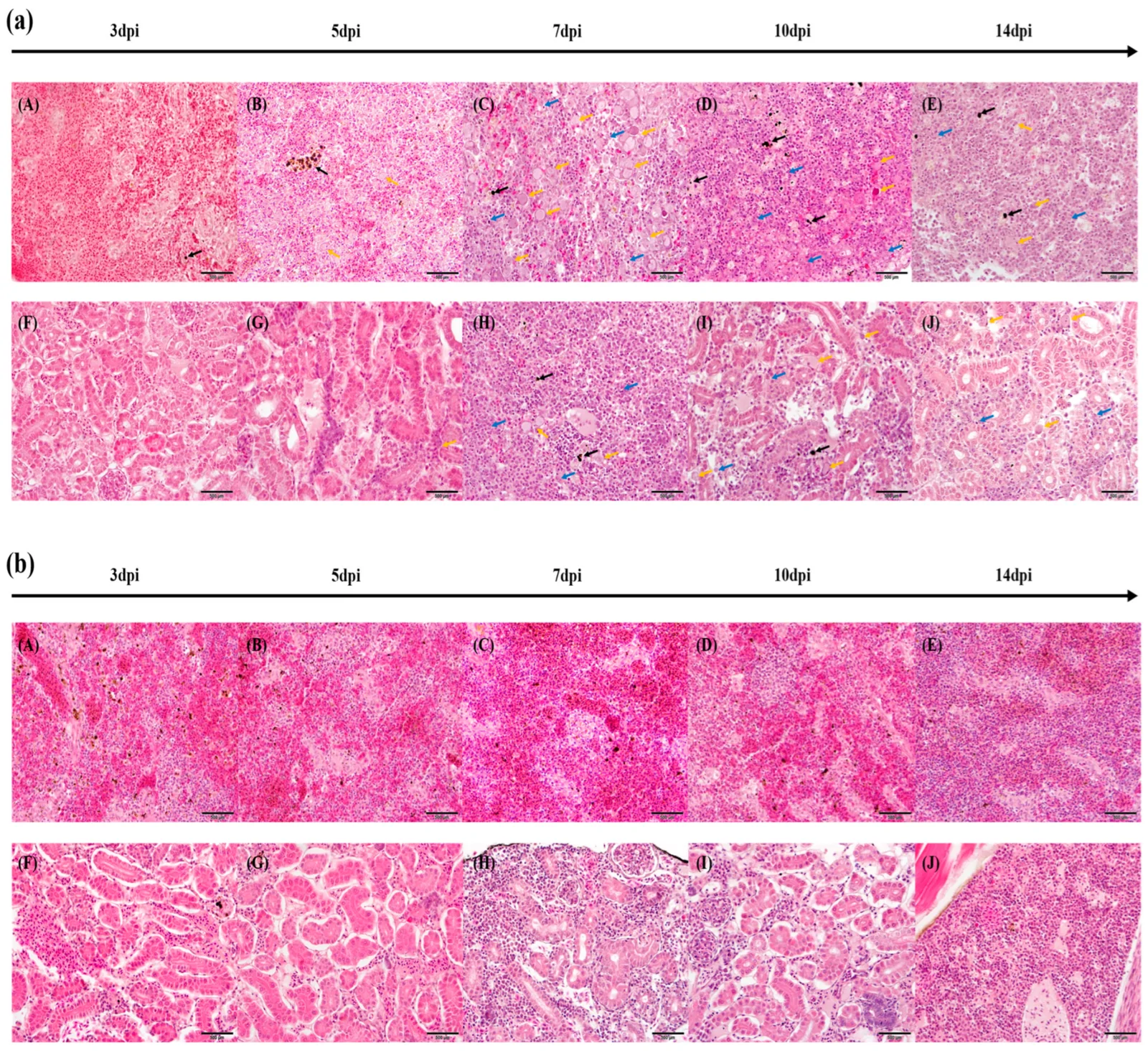
Temporal histopathological observations of the spleen and kidney in angelfish at 3, 5, 7, 10, and 14 days post-immersion (dpi) at 28 °C. (**a**) 10^5^ infectious spleen and kidney necrosis virus (ISKNV) TCID_50_/mL immersion group and (**b**) control group. Panels (**A**–**E**) and (**F**–**J**) show spleen and kidney tissues, respectively. Representative lesions include melano-macrophage centers (black arrows), enlarged basophilic cells (yellow arrows), and nuclear necrosis (blue arrows), as observed by hematoxylin and eosin (H&E) staining (scale bar = 500 μm).

**Figure 7:**
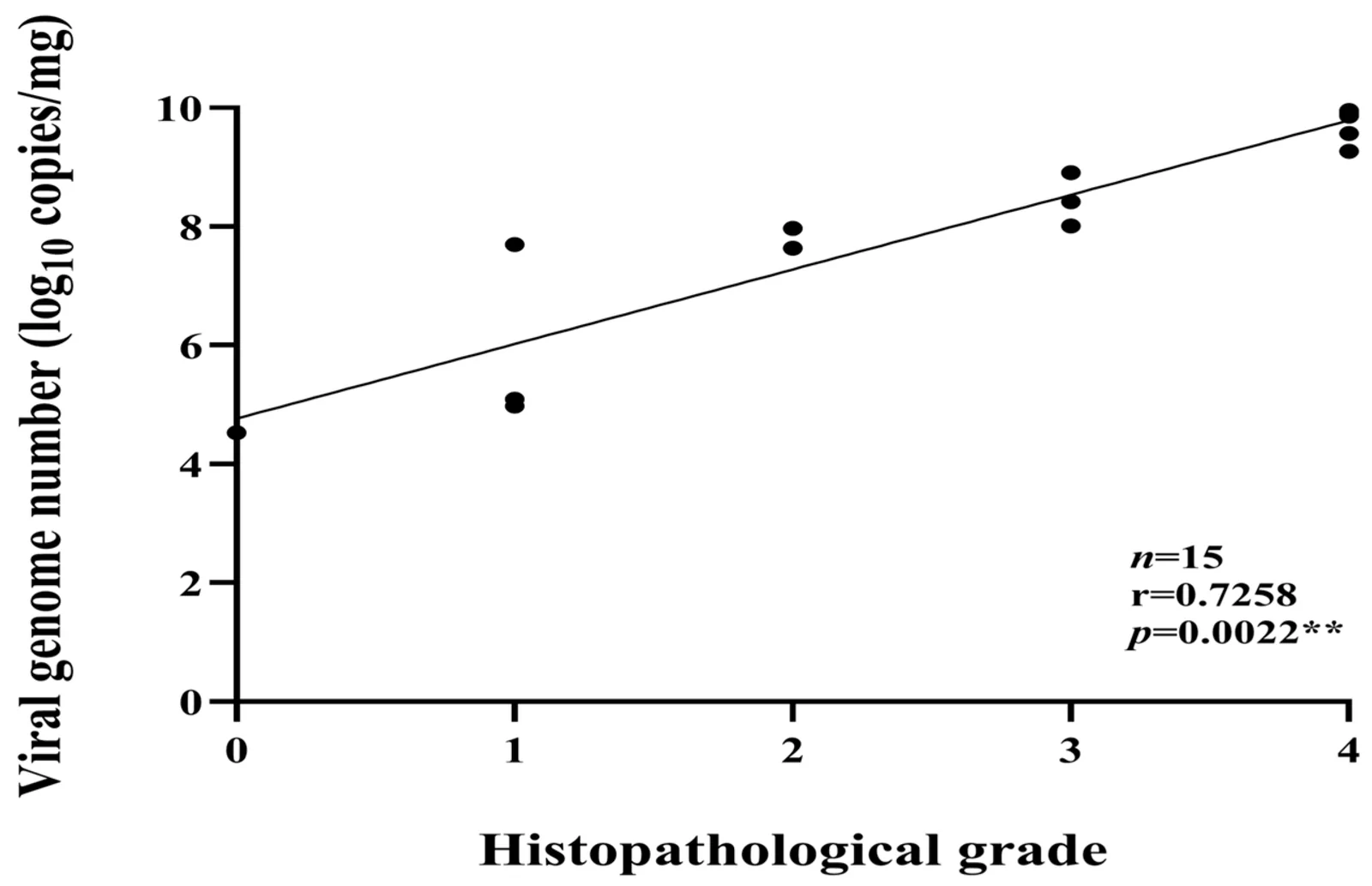
Correlation between histopathological severity and ISKNV viral load in the spleen and kidney of angelfish immersed in infectious spleen and kidney necrosis virus (ISKNV) at 10^5^ TCID_50_/mL at 28 °C. Statistical analysis significance was determined using Pearson’s correlation coefficients (r = 0.7258, 95% CI: 0.3399–0.9025, *p* < 0.01).

The correlation between viral load and histopathological severity was evaluated in the spleen and kidney of infected angelfish. At 3 dpi, activation of melano-macrophage centers (MMCs) was observed in the spleen without the presence of specific lesions typically associated with ISKNV infection, corresponding to a histopathological score of 1 (Figure 6a). At 5 dpi, hyperplastic basophilic cells appeared in the spleen and kidney, resulting in an increased histopathological score of 2. The most severe lesions were observed at 7 dpi (score 4), characterized by numerous hyperplastic basophilic cells and necrotizing lesions in spleen and kidney tissues, coinciding with the peak viral load detected (8.28 × 10^9^ copies/mg) (Figure 5 and Figure 6a). Peak viral replication coincided with maximal histopathological severity. At 10 and 14 dpi, the number of hypertrophic cells in the spleen decreased, resulting in a reduction in the histopathological grade (score 3) to mild-to-moderate grades, corresponding to a viral load of 9.78 × 10^8^–1.40 × 10^9^ copies/mg. Histopathological severity decreased in parallel with declining viral loads. Consistently, a positive correlation between viral load and histopathological grade was observed in ISKNV-immersion-infected angelfish (r = 0.7258, *p* < 0.01) (Figure 7).

### 3.4. Stage-Dependent Horizontal Transmission Following Short-Term Cohabitation

**Figure 8:**
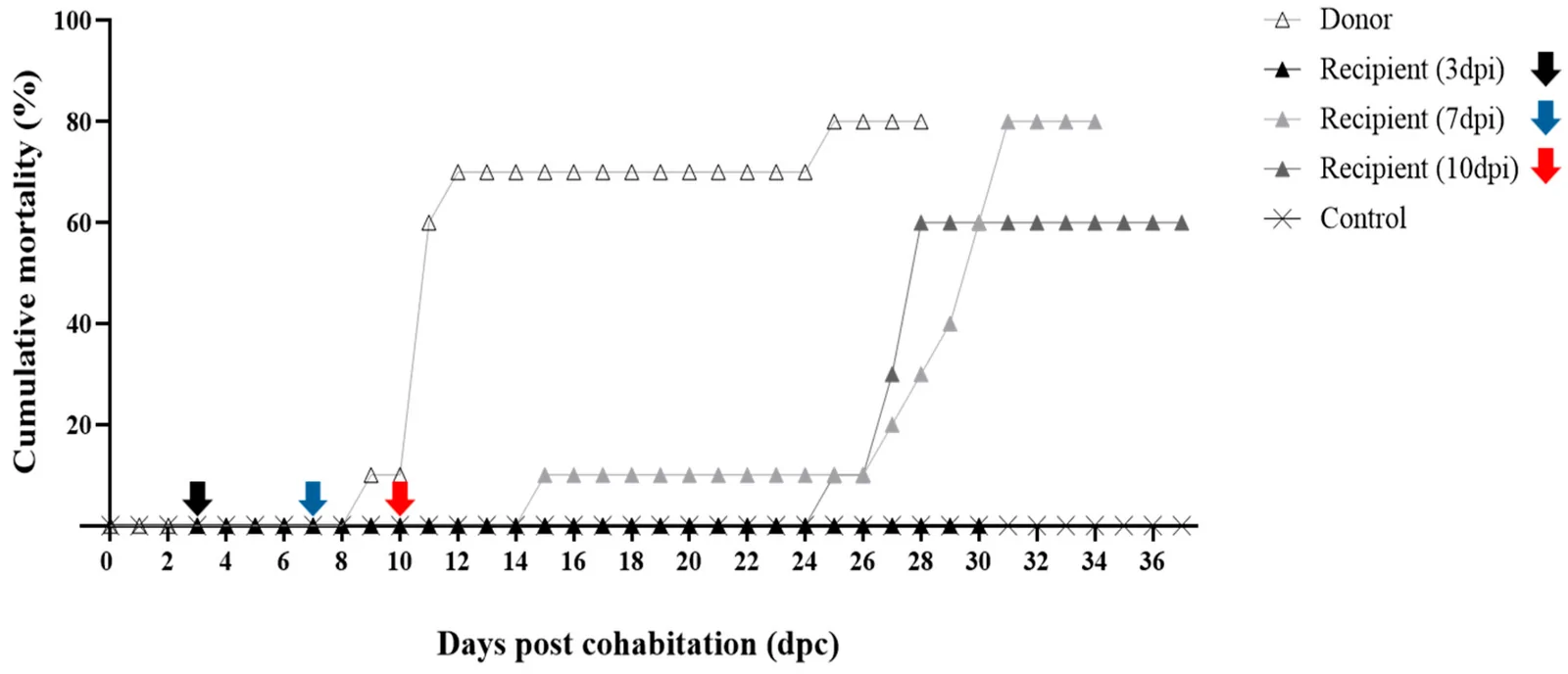
Cumulative mortality (%) of angelfish following infectious spleen and kidney necrosis virus (ISKNV) infection via cohabitation challenges. Naive recipient angelfish were cohabitated with donor fish previously immersed with 10^5^ TCID_50_/mL ISKNV for 24 h in a 10 L aquatic tank. Recipient fish were exposed at three different donor infection stages (3, 7, and 10 days post-immersion [dpi]) and subsequently monitored for cumulative mortality for 28 days post-cohabitation (dpc).

To evaluate whether ISKNV transmission occurred during short-term cohabitation, recipient angelfish were cohabited with infected donor fish for 24 h at different stages of infection (3, 7, and 10 dpi). Following short-term cohabitation and subsequent separation, cumulative mortality was observed in both donor and recipient groups. Donor fish exhibited a cumulative mortality rate of 80% by 25 dpc (days post-cohabitation). Among recipients, those cohabited with donors at 3 dpi showed no mortality up to 28 dpc. In contrast, recipients exposed to donors at 7 and 10 dpi exhibited cumulative mortality rates of 80% and 60%, respectively, with the first mortalities observed at 7 and 14 dpc, respectively. No mortality was observed in the negative control group (Figure 8).

**Figure 9:**
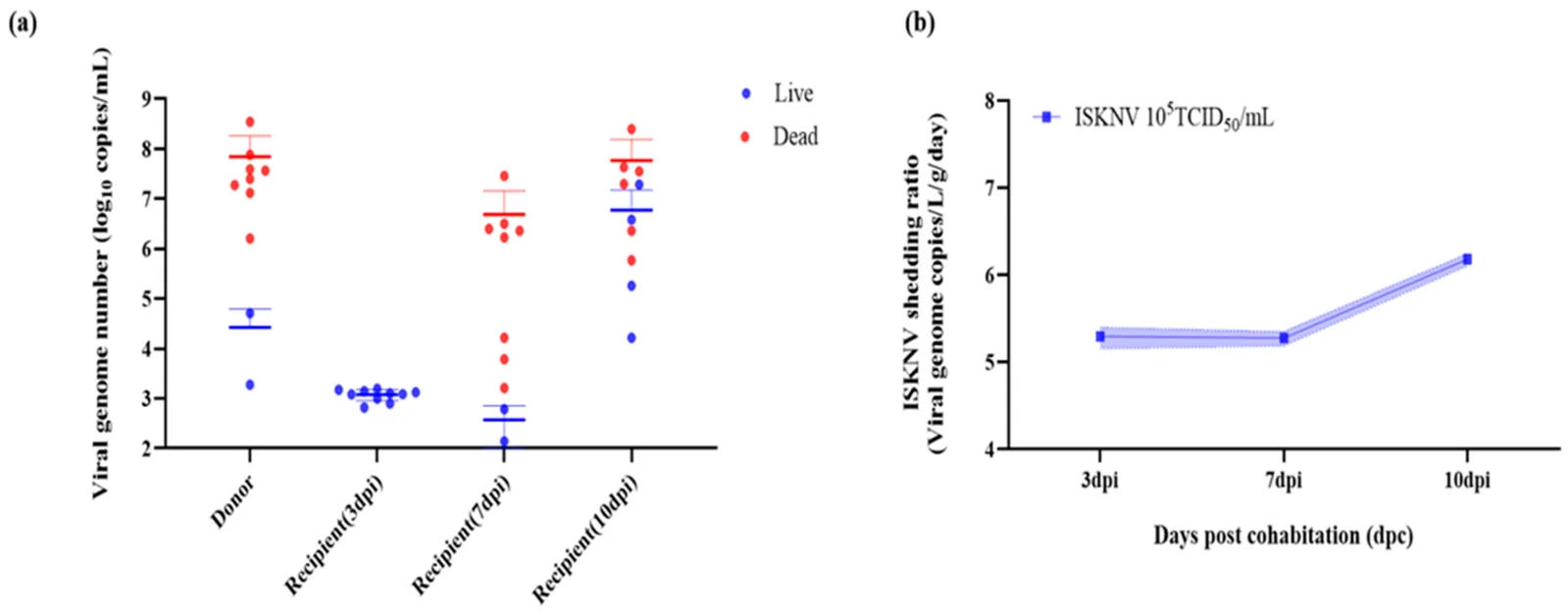
Correlation between infectious spleen and kidney necrosis virus (ISKNV) viral load in angelfish tissues and viral shedding into rearing water during short-term cohabitation. (**a**) Viral genome copy numbers detected in donor and recipient angelfish following 24 h cohabitation at 3, 7, and 10 days post-immersion (dpi). Each dot represents an individual fish, and horizontal bars indicate mean ± standard deviation (SD). (**b**) Quantification of ISKNV genome copies in rearing water following 24 h cohabitation at different donor infection stages (3, 7, and 10 dpi). Data are presented as mean ± SD of three independently processed 500 mL water subsamples collected from the same experimental tank at each donor infection stage.

To further characterize the cohabitation experiment, viral load and shedding kinetics were analyzed in both donor and recipient fish. Donor fish maintained viral loads exceeding 10^7^ ISKNV copies/mg in spleen and kidney tissues until 25 dpc, when the final mortality was recorded (Figure 9a). ISKNV transmission was confirmed in all recipient groups after 24 h of cohabitation, with viral loads ranging from low to high, depending on the infection stage of the donor fish. The recipient group cohabited with donors at 3 dpi showed no mortality; however, all surviving fish were lightly infected with ISKNV. In contrast, recipients cohabited with donors at 7 and 10 dpi showed ISKNV infection in both dead and surviving fish. Notably, the recipient group cohabited with donors at 10 dpi exhibited the highest viral load, reaching 4.42 × 10^7^ copies/mg in the spleen and kidney tissues of dead fish. Furthermore, surviving fish in this group showed substantial viral loads (>1.82 × 10^5^ copies/mg), indicating successful horizontal transmission. Quantification of ISKNV genome copies in rearing water showed higher viral shedding levels at later stages of donor infection. The highest concentration (1.63 × 10^6^ copies/L/g) was detected in water samples collected from the tank housing donors at 10 dpi (Figure 9b). Higher waterborne viral loads at 7 and 10 dpi coincided with greater cumulative mortality in the corresponding recipient groups.

## 4. Discussion

This study aimed to evaluate ISKNV infection dynamics in angelfish, a susceptible host species, and to assess the potential for stage-dependent horizontal transmission through short-term cohabitation. These findings are consistent with previous reports identifying angelfish as a naturally susceptible host species [22,23,33]. However, previous studies on the temporal progression and horizontal transmission dynamics of ISKNV in angelfish remain limited. This study therefore investigated the progression of ISKNV infection, viral shedding kinetics, and stage-dependent horizontal transmission under controlled freshwater conditions to evaluate the potential risk of transmission. Accordingly, this study (1) demonstrated the susceptibility of angelfish to ISKNV immersion infection, (2) characterized temporal viral replication and associated histopathological progression following immersion exposure, and (3) quantified viral shedding and stage-dependent horizontal transmission following short-term cohabitation.

Immersion challenge experiments demonstrated that angelfish are highly susceptible to ISKNV infection under freshwater conditions at 28 °C. Initial mortality was observed at 10^3^ TCID_50_/mL, with cumulative mortality reaching approximately 95% in the 10^6^ TCID_50_/mL immersion group. The estimated LD_50_ (%) for immersion infection was 10^4.25^ TCID_50_/mL, indicating high susceptibility of angelfish to ISKNV under immersion exposure and exhibiting an acute mortality pattern consistent with that of previously studied susceptible species [5,34,35,36,37,38]. Notably, Choi et al. [27] reported that intraperitoneal injection of the same DGIV-SAY-23 strain at 10^6^ TCID_50_/fish in pearl gourami (*Trichopodus leerii*) resulted in 100% mortality. Similarly, He et al. [35] demonstrated that juvenile mandarin fish (*S. scherzeri*) exhibited 100% cumulative mortality within 11 days following immersion exposure to 1 mL ISKNV filtrate/L under comparable temperature and exposure conditions. Thus, although cumulative mortality was limited at lower exposure concentrations, ISKNV infection was detected in a proportion of exposed angelfish. In the 10^3^ TCID_50_/mL group, 35% of fish were ISKNV-positive, and viral loads of approximately 10^4^ copies/mg were detected in surviving fish, indicating that infection could be established without substantial mortality. Furthermore, the estimated ID_50_ was 10^3.17^ TCID_50_/mL, which was approximately 10-fold lower than the LD_50_, further indicating that ISKNV infection can occur at viral concentrations below those required to induce substantial mortality. Collectively, these findings suggest that susceptibility to ISKNV infection and mortality should be considered separately, particularly under low-dose immersion exposure. Importantly, in contrast to previous studies in which the ID_50_ of ISKNV was determined using intraperitoneal injection models [39,40], this study provides experimental evidence that ISKNV infection can be successfully established through immersion exposure, highlighting the potential importance of waterborne transmission in aquaculture systems.

Distinct temporal infection dynamics and histopathological severity of ISKNV were observed following immersion infection in angelfish. No characteristic ISKNV-associated lesions were observed at 3 dpi despite detectable viral loads, likely reflecting the early stage of infection before extensive viral replication in the spleen and kidney. By 5 dpi, viral replication increased, and enlarged basophilic cells appeared in the kidney and spleen. At 7 dpi, viral load peaked at 8.28 × 10^9^ copies/mg, and the histopathological grade reached its maximum score of 4, indicating severe tissue damage. This pattern is consistent with previous studies indicating that ISKNV replication occurs optimally at temperatures between 20 and 32 °C and that the incubation period typically ranges from 7–10 days [14,35,41]. The absence of characteristic lesions at 3 dpi likely reflects limited viral replication during the initial phase of infection. In contrast, viral replication was most pronounced during the incubation period, peaking at 7 dpi under the experimental conditions of this study. Notably, the multifocal necrotic lesions and hyperplastic basophilic cells observed in the spleen and kidney at this stage closely resemble the characteristic pathological lesions of ISKNV infections described in earlier studies [5,13,18,20,34,42]. Thereafter, both viral load and histopathological scores declined at 10 and 14 dpi, with scores decreasing to 3 (moderate). This pattern is consistent with known ISKNV replication dynamics and may reflect activation of the host immune response triggered by viral invasion [43]. As the inflammatory response persists, ellipsoid structures become thickened and MMCs form in the spleen, representing histological changes associated with host defense mechanisms against viral infection [44,45].

This study demonstrated the risk of horizontal transmission of ISKNV through short-term cohabitation, depending on the infection stage of donor fish. A previous study by Kawato et al. [17] quantified the minimum and maximum RSIV loads in seawater from a red sea bream aquaculture farm as 10^2.1^ to 10^6.6^ copies/L, indicating a broad range of viral concentrations under natural conditions. Kim et al. [18] identified a threshold of 10^4^ RSIV copies/L in seawater as the minimum concentration required for successful transmission and reported that higher viral loads significantly increased the probability of infection. Similarly, following only 24 h of cohabitation with ISKNV-infected donors, the viral load in tank water reached approximately 1.96 × 10^5^ copies/L/g by 3 dpi and continued to increase, peaking at 1.63 × 10^6^ copies/L/g at 10 dpi. These viral shedding levels exceeded previously suggested thresholds for facilitating viral transmission, implying that the ISKNV released from the donor fish during the acute infection phase was sufficient to infect naive recipients. This finding suggests that even brief cohabitation under freshwater conditions can result in significant environmental viral loads, thereby facilitating efficient ISKNV dissemination.

Moreover, higher waterborne viral loads coincided with increased viral loads in donor fish, suggesting a potential relationship between infection progression and viral shedding. Previous studies have demonstrated horizontal transmission of RSIV through cohabitation with infected donor fish [18] and have shown that viral shedding is associated with host viral load and the stage of infection [18,46]. These findings highlight the potential importance of waterborne viral shedding in horizontal transmission. In the present study, cumulative mortality rates of 80% and 60% were observed in recipients following 24 h cohabitation with donors at 7 and 10 dpi, respectively, whereas no mortality occurred in recipients exposed at 3 dpi. The recipient mortality coincided with higher levels of viral shedding at the later stages of donor infection, suggesting a potential association between donor infection stage, viral shedding, and transmission outcomes. This observation corresponds to the findings of Rimmer et al. [5], who reported that viral shedding into water is greatest during the peak stage of infection, thereby increasing the potential for horizontal transmission of ISKNV. Tropical freshwater fish constitute a major component of the global ornamental fish market, and disease outbreaks remain an important challenge for ornamental fish production [47]. Notably, this study suggests that ISKNV transmission is associated with the infection stage of donor fish and can occur following short-term exposure and may vary according to the infection stage of donor fish, highlighting the potential relevance of ISKNV transmission dynamics to disease management in the ornamental fish industry.

Several limitations of the present study should be considered. The temporal infection experiment and each cohabitation treatment were conducted using a single experimental tank, and individual fish and water subsamples collected from the same tank therefore did not represent independent experimental replicates. Accordingly, the temporal changes in viral load and histopathological lesions and the differences in transmission outcomes among donor infection stages should be interpreted within the specific experimental conditions examined. Further studies incorporating independent tank replication and additional experimental conditions are required to confirm the reproducibility of the observed infection dynamics and stage-dependent transmission findings.

## 5. Conclusions

This study demonstrated that angelfish are highly susceptible to ISKNV infection following freshwater immersion exposure, resulting in high cumulative mortality, extensive viral replication, and severe histopathological lesions in the spleen and kidney. Temporal analyses revealed that viral replication and lesion severity peaked during the acute phase of infection, indicating a close association between viral kinetics and pathological progression. Furthermore, infected fish released substantial quantities of viral genomes into the surrounding water, and ISKNV transmission was detected following only 24 h of cohabitation, with recipient mortality and viral load varying among the donor infection stages. Overall, these findings demonstrate that the infection stage of donor fish is associated with viral shedding and horizontal transmission of ISKNV in angelfish under the experimental conditions examined. The occurrence of transmission following short-term cohabitation may have potential relevance to disease management in the ornamental fish industry.

## Figures and Tables

**Table 1:** Primers and probes used in this study.

Target Gene	Purpose	Sequence (5′–3′)	Conditions	Amplicon (bp)	Reference
*Pst* I fragment	Detection	F: CTC AAA CAC TCT GGC TCA TC	95 °C, 5 min; (94 °C, 30 s; 58 °C 1 min, 72 °C 1 min) × 30 72 °C, 7 min	570	[26]
		R: GCA CCA ACA CAT CTC CTA TC			
MCP	Viral quantification	F: CCA GCA TGC CTG AGA TGG A	95 °C, 10 min; (94 °C, 10 s; 60 °C, 35 s) × 40	128	[30]
		R: GTC CGA CAC CTT ACA TGA CAG G			
		P: FAM-TAC GGC CGC CTG TCC AAC G-BHQ1			

## Data Availability

The data presented in this study are available upon request from the corresponding author.

## Supplementary Materials

The following supporting information can be downloaded at: https://www.mdpi.com/article/10.3390/ani16172751/s1, Figure S1: Polymerase chain reaction (PCR)-based confirmation of infectious spleen and kidney necrosis virus (ISKNV) infection in angelfish. The representative viral infection was confirmed by PCR using the RSIV/ISKNV-specific primer set (1F/1R, 570bp) by PCR. Agarose gel electrophoresis showing amplification followed by Pst-I restriction fragment gene from spleen and kidney tissues collected from angelfish immersed in graded concentrations of DGIV-SAY-23 (102 to 106 TCID50/mL). The experiment was conducted in two independent biological replicates, yielding consistent results. No amplified bands were detected in the negative control group. M, DNA marker; –ve, negative control; +ve, positive control.

## Abbreviations

Abbreviations used in this manuscript are as follows:

ISKNV	Infectious spleen and kidney necrosis virus
RSIV	Red sea bream iridovirus
DGF	Dwarf gourami fin
CPE	Cytopathic effect
ATPase	Adenosine triphosphatase
MCP	Major capsid protein
PCR	Polymerase chain reaction
L-15	Leibovitz’s
FBS	Fetal bovine serum
NEAA	Non-essential amino acids
HEPES	Hydroxyethyl piperazine ethane sulfonic acid
TCID_50_	Median tissue culture infectious dose
PBS	Phosphate-buffered saline
DPI	Days post-immersion
DPC	Days post-cohabitation
