# Supplementary material for: Shedding Kinetics and Infection Stage-Dependent Horizontal Transmission of Infectious Spleen and Kidney Necrosis Virus (ISKNV) in Angelfish (Pterophyllum scalare)
Source: Animals (Basel). 2026 Sep 2;16(17):2751. doi: 10.3390/ani16172751 (PMC13565678; doi:10.3390/ani16172751)
Supplement: Supplementary file 1 [file animals-16-02751-s001.zip › animals-4495969-supplementary.pdf]

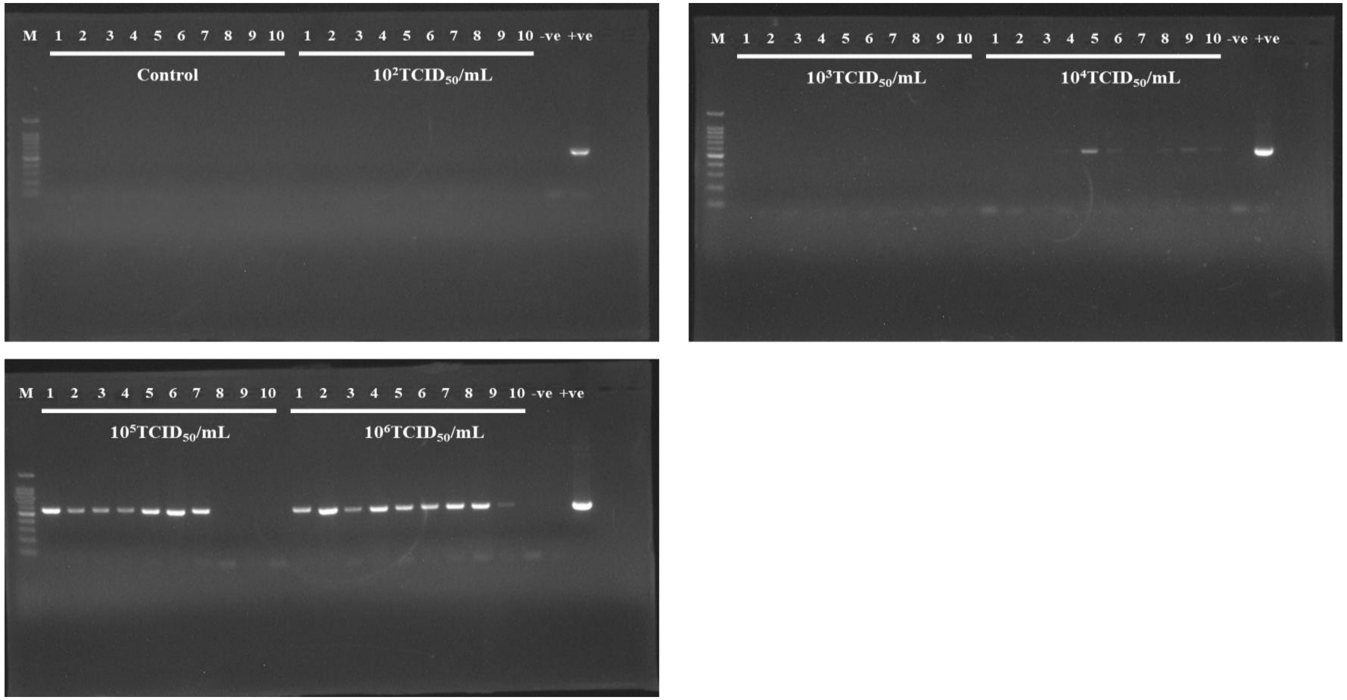

Supplemental Figure S1. Polymerase chain reaction (PCR)-based confirmation of infectious spleen and kidney necrosis virus (ISKNV) infection in angelfish. The representative viral infection was confirmed by PCR using the RSIV/ISKNV-specific primer set (1F/1R, 570bp) by PCR. Agarose gel electrophoresis showing amplification followed by *Pst-I* restriction fragment gene from spleen and kidney tissues collected from angelfish immersed in graded concentrations of DGIV-SAY-23 (10^2 to 10^6 TCID$_{50}$/mL). The experiment was conducted in two independent biological replicates, yielding consistent results. No amplified bands were detected in the negative control group. M, DNA marker; –ve, negative control; +ve, positive control.
